# Funders' Expectations for Open Science in Cardiovascular Research: A Scoping Review of the Largest Cardiovascular Research Funders

**DOI:** 10.1161/JAHA.125.048584

**Published:** 2026-05-06

**Authors:** Anna Catharina Vieira Armond, Al Mamoune Alaoui, David Moher, Jean Rouleau, Kelly D. Cobey

**Affiliations:** ^1^ University of Ottawa Heart Institute Ottawa ON Canada; ^2^ Centre for Journalology, Clinical Epidemiology Program Ottawa Hospital Research Institute Ottawa ON Canada; ^3^ School of Epidemiology and Public Health, Faculty of Medicine University of Ottawa Ottawa ON Canada; ^4^ Montreal Heart Institute University of Montreal Montreal QC Canada

**Keywords:** data management, health policy, open access publishing, open science, patient participation, policy, reproducibility of results, Epidemiology, Ethics and Policy

## Abstract

Open science practices, including data sharing, open access, and prospective study registration, have been increasingly recognized to improve transparency, reproducibility, and accessibility in research, yet their uptake and implementation by cardiovascular research funders is unclear. We conducted a scoping review of publicly available policies, guidance, and grant instructions from 12 members of the Global Cardiovascular Research Funders Forum to assess expectations, monitoring, and support for open science in cardiovascular research. We included 105 documents from 9 funders; no relevant documents were identified for 3 funders. Data sharing (67%) and open access (58%) were the most common mandates by funders, followed by prospective registration (50%) and patient and public involvement (50%). Requirements for other practices, including code sharing, use of reporting guidelines, preprints, and open peer review, were uncommon. Monitoring compliance was inconsistent, with many funders not specifying any mechanisms, even for widely required practices. Where available, support was most often provided through financial assistance, guidance, or infrastructure, particularly for open access, data sharing, and patient and public involvement. These findings suggest that while cardiovascular funders are engaging with open science, policies remain uneven in scope, monitoring, and support. Coordinated efforts to strengthen and harmonize open science expectations, particularly around compliance monitoring and researcher training, will be essential to realizing the full potential of open science in cardiovascular research.

**Nonstandard Abbreviations and Acronyms**
ORCID: Open Researcher and Contributor ID
PPI: patient and public involvement

Open science refers to a broad set of practices that aim to make scientific research more transparent, accessible, collaborative, and reproducible. While there is no single consensus on what open science entails, the practices include, but are not limited to, sharing research data, code, and materials; prospectively registering studies; publishing in open access; using reporting guidelines; and engaging in open peer review.^1^ Globally, open science has gained momentum as a strategy to foster research integrity, collaboration, and accelerate scientific discovery across disciplines, including biomedical research.^1^, ^2^ Cardiovascular research, like many other fields, faces growing demands to improve the transparency and reproducibility of its research outputs.^3^ Prior audits show that most cardiology publications do not make data, protocols, or statistical analysis scripts publicly available,^4^ and present inconsistent reporting,^5^ echoing broader concerns about research waste and reproducibility.^6^, ^7^ Open science practices offer a potential solution to these challenges, but their uptake remains variable^8^ and implementation is often influenced by institutional and systemic factors, including the policies and expectations of research funders. These issues are especially pressing given the global burden of cardiovascular disease and the imperative to translate research findings efficiently into clinical care.^9^

Funders play a critical role in shaping research practices by setting requirements and providing incentives for grant recipients. By mandating or recommending open science practices, funders can promote a culture of transparency and reproducibility. Most previous analyses have focused on general biomedical funders or specific practices, such as data sharing,^10^ often neglecting the cardiovascular research landscape as a distinct domain with its own needs and challenges. For example, an international survey of 198 cardiovascular researchers^8^ reported that many participants had limited formal training in open science: 52% learned largely on the job, and 36% had received no training at all. Respondents indicated that additional funding and institutional support were critical to adopting open science practices, with funders seen as key interest holders to influence their behaviors. Little is known about how cardiovascular research funders are addressing open science in their policies and guidance and to what degree there is coordination internationally across cardiovascular funders.

To address this gap, we conducted a scoping review to identify and assess publicly available policies and guidance related to open science practices among funders who are members of the Global Cardiovascular Research Funders Forum, a global partnership of 12 major cardiovascular research funders.^11^ Our objectives were to (1) map the extent to which open science practices are addressed in these funders' official documents, (2) classify their expectations (eg, mandated, recommended), and (3) assess the types of support and monitoring mechanisms provided. By synthesizing this information, we aim to inform future efforts to align open science policies across funders and support their implementation in cardiovascular research.

## Methods

All data and materials underlying this study are publicly available on the Open Science Framework at https://doi.org/10.17605/OSF.IO/9JEFW.

### Protocol and Registration

We conducted a scoping review to identify and analyze publicly available policies and guidance related to open science practices from cardiovascular research funders. This review followed the Joanna Briggs Institute's methodology for scoping reviews and is reported in accordance with the Preferred Reporting Items for Systematic Reviews and Meta‐Analyses Extension for Scoping Reviews.^12^ The study protocol was registered prospectively (https://doi.org/10.17605/OSF.IO/9PZTA).

### Eligibility Criteria

We included documents that addressed at least 1 open science practice (eg, open access, open data, prospective registration, preprints) and were publicly available from cardiovascular research funders. Eligible document types included official policies, guidance documents, grant requirements, application instructions, and relevant web pages. Documents were included if written in English or if a reliable translation could be produced using DeepL Translate. Opinion pieces, blogs, and nonofficial funder communications were excluded.

### Information Sources

We focused on members of the Global Cardiovascular Research Funders Forum, an international coalition of 12 major cardiovascular research funders:

1. American Heart Association (United States)
2. British Heart Foundation (United Kingdom)
3. Danish Heart Foundation
4. Dutch Heart Foundation (Hartstichting)
5. German Centre for Cardiovascular Research (DZHK)
6. Heart and Stroke Foundation of Canada
7. Institute of Circulatory and Respiratory Health (Canadian Institutes of Health Research, Canada)
8. Leducq Foundation
9. National Heart Foundation of Australia
10. National Heart Foundation of New Zealand
11. National Heart, Lung, and Blood Institute (National Institutes of Health, United States)
12. Swiss Heart Foundation

We defined open science as an umbrella term encompassing practices that promote transparency, reproducibility, accessibility, and collaboration in research. Documents were included if they referred to at least 1 of the following practices: open access, open data, data management, open code, open materials, prospective registration, transparent reporting, reproducibility practices, preprints, citizen science (including patient and public involvement [PPI]), open peer review, or researcher identifiers (eg, Open Researcher and Contributor ID [ORCID]). Table 1 presents the descriptions of each of the investigated practices.

**Table 1:** Open Science Practices Descriptions

Open science practice	Description
Open access	The practice of making scholarly work freely available online, allowing anyone to access, read, and use it for lawful purposes without financial, legal, or technical barriers
Data sharing	The practice of making the underlying data sets used to generate research findings accessible to other researchers, policymakers, and the public, either openly or under controlled conditions
Code sharing	Code sharing in science refers to the practice of making the scripts, software, or computational workflows used in a study openly available to others. Sharing code allows peers to reproduce analyses, validate findings, and adapt existing methods for new research questions
Prospective registration	The practice of publicly registering a study's protocol, including research questions, outcomes, design, and planned analyses, before data collection or examination
Preprint	Preprints refer to scientific manuscripts or research findings that are made publicly available in advance of formal publication, typically referred to as a form of green open access
Public and patient involvement	Public and patient involvement refers to the active partnership of patients, caregivers, and members of the public in the research process. Rather than being the subjects of research, they contribute to shaping research priorities, study design, conduct, and dissemination
Data management plans	Research data management involves the systematic organization, storage, documentation, and preservation of research data throughout its life cycle. Data management plans are formal documents outlining how data will be collected, managed, shared, and preserved
Open peer review	Open peer review is an umbrella term for peer review models that aim to increase transparency in the evaluation of scholarly work. It can include practices such as revealing reviewer identities, publishing review reports alongside articles, allowing public comments, or enabling authors and reviewers to interact directly
Use of ORCID	Using a unique digital identifier for researchers that links their professional activities, publications, and data sets. ORCID enhances transparency, ensures proper attribution, and facilitates tracking of contributions across projects and platforms
Rigor and reproducibility	Ensuring that research is conducted with robust, transparent, and unbiased methods so that results can be independently verified. Rigor refers to the strict application of scientific principles and methods, while reproducibility means that findings can be consistently obtained when experiments or analyses are repeated under the same conditions
Material sharing	Material sharing refers to the practice of making physical research resources, such as biological samples, cell lines, reagents, instruments, or other study materials, available to other researchers
Use of reporting guidelines	Tools or instructions designed to help authors transparently report research using explicit methods. These can take the form of checklists, flowcharts, or structured text to ensure clarity, completeness, and consistency in reporting

ORCID indicates Open Researcher and Contributor ID.

We restricted our analysis to cardiovascular research funders and included only documents available on official websites, without date restrictions.

### Search Strategy

Two reviewers independently conducted searches between January and April 2025. Searches were performed on funders' websites using internal search tools and supplemented with Google queries combining funder names with targeted keywords (eg, “open science,” “open data,” “data sharing,” “preprints,” “preregistration,” “reporting guideline,” “ORCID,” “patient involvement,” “open access,” and “reproducibility”). The first 100 Google hits per funder were screened for relevance.

### Data Extraction and Analysis

We developed a customized data extraction form and piloted it on 5 documents to ensure clarity and consistency. One reviewer extracted all data, and a second reviewer conducted quality control on all records. Discrepancies were resolved by consensus or with input from a third reviewer when necessary. Data extraction was performed using Airtable, a cloud‐based database platform.

We extracted the following information:

1. Funder name and country
2. Document type (eg, policy, grant call, guidance)
3. Year of publication or last update
4. Cardiovascular research focus (if specified)
5. Open science practices mentioned
6. Whether practices were required, recommended, mentioned without further detail, or not mentioned
7. Monitoring or compliance mechanisms
8. Forms of support (eg, training, financial resources, infrastructure)

Some funders developed multiple documents corresponding to different funding opportunities, which varied in their expectations for open science practices. For example, 1 funder's clinical trial program explicitly required data sharing, while its early‐career investigator program only recommended it. These documents also differed in scope, with some providing a high‐level perspective on open science and others outlining specific policies for a certain practice.

To ensure consistency and synthesize information from multiple documents at the funder level, we used an inclusive coding approach and applied an ordinal coding reflecting the level of stringency of each open science practice: (1) “required,” (2) “recommended,” (3) “mentioned but not discussed,” and (4) “not mentioned.” Coding was first conducted at the document level. We then summarized each funder per practice by selecting the most stringent level observed across all eligible documents (the minimum value on the ordinal scale). For example, if a practice was required in at least 1 document but only recommended or not mentioned in others, it was classified as “required” at the funder level.

The same approach was applied to the assessment of monitoring and support mechanisms for open science practices. When multiple documents from a funder described monitoring or support, we summarized the funder by keeping the most comprehensive level of information reported across documents.

This approach reflects the presence of at least 1 formal requirement or mechanism within the funder's policy landscape.

Quantitative data were summarized descriptively using counts and proportions. For qualitative data, we conducted a thematic analysis following the approach by Braun and Clarke.^13^ Two reviewers independently reviewed and coded the extracted text, refined the codes through discussion, and organized them into overarching themes. Any disagreements were resolved with input from an additional reviewer. Thematic synthesis was conducted using Microsoft Excel (Research Resource Identifier: SCR_016137).

## Results

**Figure 1:**
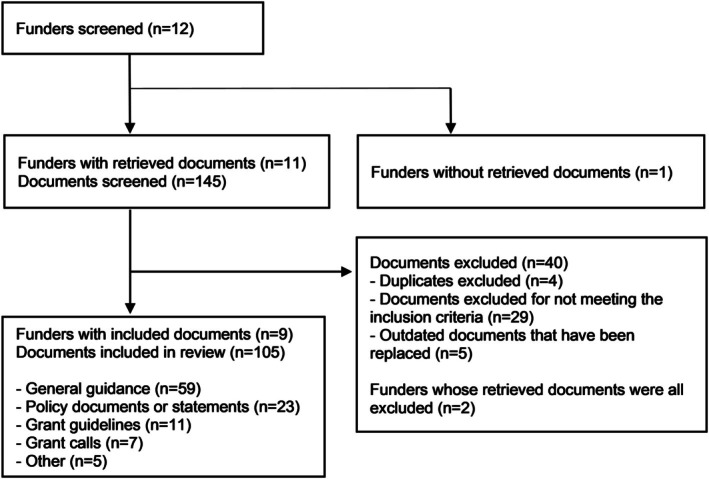
Flowchart of funders and documents included in the scoping review.

We identified 145 documents from 11 (of the 12) cardiovascular research funders across 9 countries in the Global Cardiovascular Research Funders Forum based on searches on the funders' websites and Google. For 1 funder, no potentially relevant documents could be located. After duplicates were removed, a total of 142 documents were assessed for eligibility. Of these, 38 documents were excluded for not meeting the inclusion criteria (eg, documents lacked relevance to open science expectations or did not pertain to research funding). This resulted in 105 documents from 9 funders included in this review (Figure 1). The 3 funders for whom we did not locate relevant documents were the Leducq Foundation, the National Heart Foundation of New Zealand, and the Swiss Heart Foundation.

Most documents were classified as general guidance or instructions (59 [56%]), followed by policy or formal statement (23 [22%]), grant guidelines (11 [10%]), grant calls (7 [7%]), and other (5 [5%]). Where available, most documents were published or updated in 2024.

### Open Science Expectations

**Figure 2:**
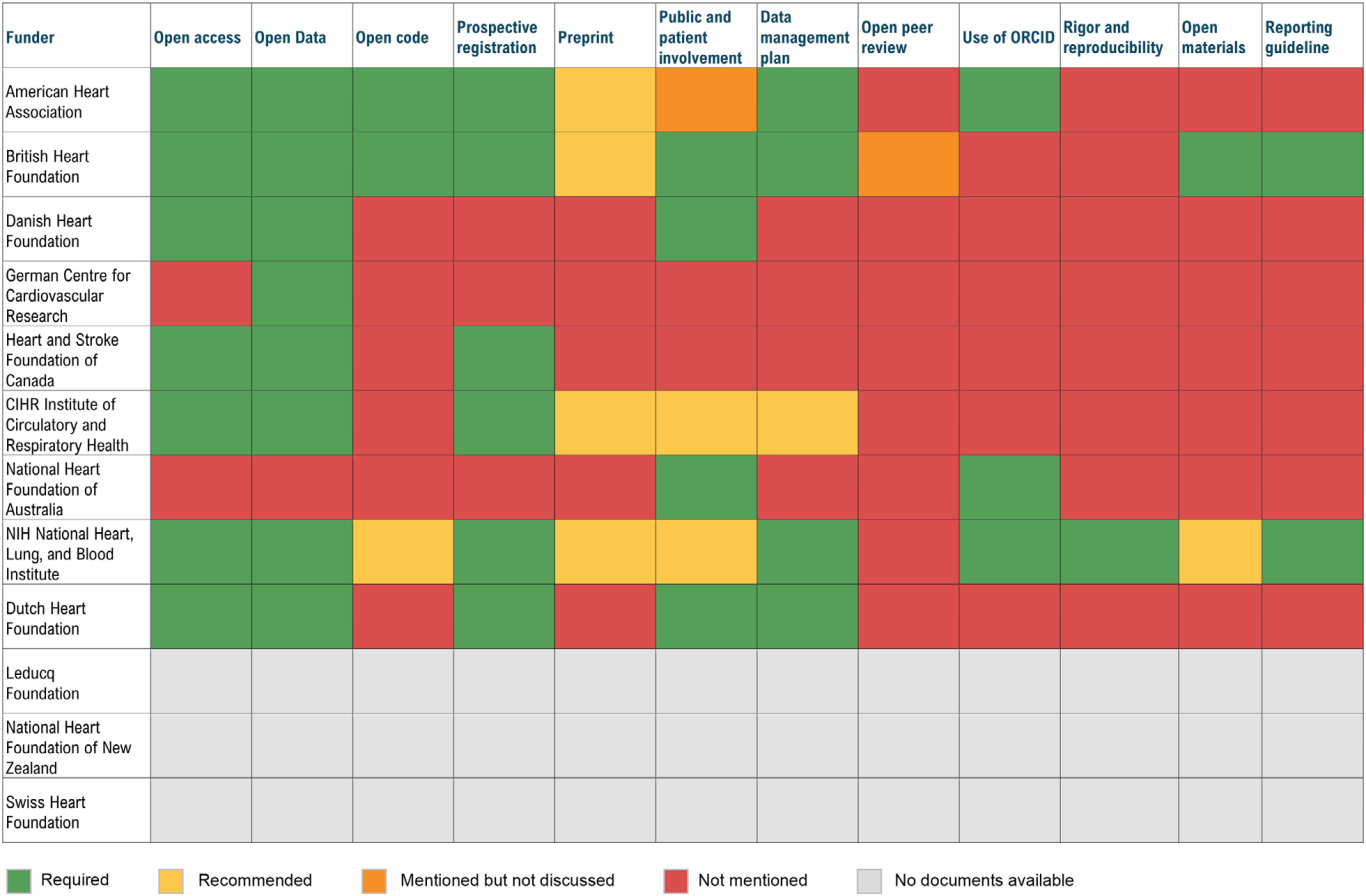
Funders' expectations for open science practices. CIHR indicates Canadian Institutes of Health Research; NIH, National Institutes of Health; and ORCID, Open Researcher and Contributor ID.

We investigated 12 cardiovascular research funders for their expectations on open science practices. To calculate the proportion of funders addressing each open science practice, all 12 funders were included in the denominator. However, the 3 funders without any documents were not assigned classifications (eg, required, recommended, or not mentioned). The extent to which various practices were required, recommended, mentioned, not mentioned, monitored, or supported is presented in Figure 2.

The most commonly required practices were data sharing (8 funders [67%]) and open access (7 funders [58%]), followed by prospective registration (6 funders [50%]). Data management plans were required by 4 funders (33%) and recommended by 1 (8%). Similarly, PPI was required by 4 funders (33%) and recommended by 2 (17%). Use of ORCID identifiers was required by 3 funders (25%), and code sharing was required by 2 funders (17%) and recommended by 1 (8%). Use of reporting guidelines was required by 2 funders (17%), and open materials were required by 1 (8%) and recommended by 1 funder (8%) (Figure 2).

Preprints were not required by any funders but were recommended by 4 (33%). Open peer review was rarely addressed, mentioned by only 1 funder (8%). Rigor and reproducibility were explicitly required by just 1 funder (8%).

When restricting the denominator to funders with retrievable eligible documents (n=9), the proportion of funders addressing each open science practice was higher (see Table S1).

### Monitoring and Support of Open Science Practices

The extent to which funders monitored compliance with open science expectations varied across practices. In some cases, monitoring mechanisms focused on verifying whether required elements (eg, patient involvement plans) were included in project proposals rather than assessing their implementation during the project. Among funders that required or recommended a given open science practice, monitoring mechanisms were inconsistently reported (Table 2).

**Table 2:** Monitoring of Open Science Practices by Cardiovascular Research Funders

Open science practice	Funders for whom the practice is required or recommended, n	Compliance monitoring		
		Yes, n (%)	Mentioned but not discussed, n (%)	Not mentioned, n (%)
Data sharing	8	2 (25)	2 (25)	4 (50)
Open access	7	2 (29)	4 (57)	1 (14)
Prospective registration	6	3 (50)	1 (17)	2 (33)
Public and patient involvement	6	3 (50)	1 (17)	2 (33)
Data management plans	5	3 (60)	0 (0)	2 (40)
Preprint	4	0 (0)	0 (0)	4 (100)
Code sharing	3	1 (33)	0 (0)	2 (67)
Use of ORCID	3	3 (100)	0 (0)	0 (0)
Material sharing	2	0 (0)	0 (0)	2 (100)
Use of reporting guidelines	2	0 (0)	0 (0)	2 (100)
Rigor and reproducibility	1	1 (100)	0 (0)	0 (0)
Open peer review	0	0 (0)	0 (0)	0 (0)

Percentages are based on the total number of funders for which the practice was required or recommended. ORCID indicates Open Researcher and Contributor ID.

For data sharing, 2 of 8 funders (25%) reported monitoring, 2 (25%) mentioned monitoring without further detail, and 4 (50%) did not mention monitoring. For open access, 2 of 7 funders (29%) indicated that compliance was monitored, 4 (57%) mentioned monitoring without further detail, and 1 (14%) did not mention monitoring. Monitoring was more frequent for data management plans (3/5 [60%]), ORCID use (3/3 [100%]), and rigor and reproducibility requirements (1/1 [100%]). Prospective registration and PPI were monitored by half of the relevant funders (3/6 [50%]), whereas monitoring of code sharing was reported by 1 of 3 funders (33%). No monitoring mechanisms were identified for preprints, material sharing, or the use of reporting guidelines. Support mechanisms also varied widely across practices (Table 3). Financial support to cover related costs, either through dedicated funding, fee reimbursements, or by allowing these costs to be included in grant budgets, was most frequently offered for open access (86%), data management (80%), data sharing (50%), and PPI (50%), reflecting the resource demands of these practices. One funder (14%) explicitly stated that it does not cover article processing charges.

**Table 3:** Support Provided by Cardiovascular Research Funders for Each Open Science Practice

Open science practice	Funders for whom the practice is required or recommended, n	Financial support			Implementation support			
		Costs covered, n (%)	Costs not covered, n (%)	Not mentioned, n (%)	Instructions or guiding materials, n (%)	Infrastructure, n (%)	Formal or informal training, n (%)	Not mentioned, n (%)
Data sharing	8	4 (50)	0 (0)	4 (50)	3 (38)	3 (38)	1 (13)	4 (50)
Open access	7	6 (86)	1 (14)	0 (0)	3 (43)	2 (29)	1 (14)	3 (43)
Prospective registration	6	1 (17)	0 (0)	5 (83)	4 (67)	0 (0)	1 (17)	2 (33)
Public and patient involvement	6	3 (50)	0 (0)	3 (50)	5 (83)	1 (17)	2 (33)	1 (17)
Data management plans	5	4 (80)	0 (0)	1 (20)	4 (80)	0 (0)	2 (40)	1 (20)
Preprint	4	0 (0)	0 (0)	4 (100)	3 (75)	0 (0)	0 (0)	1 (25)
Code sharing	3	0 (0)	0 (0)	3 (100)	1 (33)	1 (33)	0 (0)	1 (33)
Use of ORCID	3	0 (0)	0 (0)	3 (100)	1 (33)	0 (0)	0 (0)	2 (67)
Material sharing	2	0 (0)	0 (0)	2 (100)	0 (0)	0 (0)	0 (0)	2 (100)
Use of reporting guidelines	2	0 (0)	0 (0)	2 (100)	1 (50)	0 (0)	0 (0)	1 (50)
Rigor and reproducibility	1	0 (0)	0 (0)	1 (100)	1 (100)	0 (0)	1 (100)	0 (0)
Data sharing	8	4 (50)	0 (0)	4 (50)	3 (38)	3 (38)	1 (13)	4 (50)

Percentages are based on the total number of funders for which the practice was required or recommended. ORCID indicates Open Researcher and Contributor ID.

Funders also provide implementation support, such as training, infrastructure, or guiding materials. Guiding materials were widely provided across several practices, including open access (43%), data sharing (38%), prospective registration (67%), and data management plans (80%). Infrastructure included platforms or resources that facilitate implementation, such as data repositories, patient engagement platforms to identify patient partners, or systems that automatically make an article open access via PubMed Central. Infrastructure was provided for data and code sharing (38% and 33%, respectively), open access (29%), and PPI (17%). Formal or informal training was less commonly reported overall but was most commonly provided for data management plans (40%), PPI (33%), and prospective registration (17%).

## Discussion

This scoping review provides a comprehensive assessment of open science expectations among 12 major cardiovascular research funders globally. Our findings show that while many funders have incorporated some key open science practices, formal requirements remain unevenly applied across the range of open science practices. Notably, data sharing and open access were the most frequently required practices (67% and 58%, respectively), reflecting broad recognition of their central role in fostering accessibility and transparency of science. The creation of data management plans was also required or recommended by nearly half of the funders (42%). As structured frameworks for planning data collection, storage, and sharing, data management plans operationalize the commitment to data sharing and reproducibility.

In addition, practices such as PPI and prospective registration were required or recommended by half of the funders, suggesting a growing emphasis on patient‐centered research and research transparency. The increasing attention to PPI reflects its recognized importance across clinical practice, guideline development, and research, particularly in cardiovascular research, where patient organizations, scientific societies, and major funders have increasingly advocated for its integration.^14^, ^15^

Beyond patient involvement, prospective registration expectations also illustrate how policy development may be shaped by existing regulatory frameworks. While most funders required prospective registration for clinical trials, it was often only recommended or not mentioned for other types of studies. This pattern likely reflects the longer‐standing regulatory and ethical frameworks governing clinical trials, where prospective registration is widely recognized as essential to reduce selective reporting and publication bias.^16^ In contrast, expectations for other study designs, such as observational studies or secondary analyses, remain less consistently defined.

This variability has important implications for how funder policies are interpreted and applied. Requirements that apply only to specific study types or funding mechanisms may not be apparent to applicants navigating different programs and can contribute to inconsistent adoption of open science practices. More broadly, this example also reflects a challenge in open science policy development: ensuring that expectations are specific enough to be meaningful yet consistent enough to promote transparency across all study types. Prospective registration reduces bias and increases transparency by requiring researchers to declare their hypotheses, design, and analysis plan before outcomes are known.^17^ Expanding its use to other study designs, such as observational studies and secondary analyses, could contribute to improving the reproducibility and integrity of a broader range of cardiovascular research outputs.

However, open science encompasses a broader set of practices than those currently prioritized by funders. Practices such as code sharing, use of reporting guidelines, open peer review, and preprints received far less attention, suggesting that funder policies have yet to fully reflect the scope of what transparent and reproducible research requires.

The gaps in policy scope are further reflected in how funders approach compliance: Monitoring of compliance with open science policies was inconsistent and often lacking, even for widely required practices. Without (continuous) monitoring, it is likely difficult for funders to use data to better disseminate and implement open science mandates and recommendations among their grantees. For example, while data sharing and open access are among the most frequently required practices, monitoring is only sporadically implemented; fewer than one third of funders with requirements for open access publishing or data sharing actively monitor adherence. Monitoring was somewhat more common for data management plans (60%), the use of ORCID (100%), PPI, and prospective registration (50%), which may reflect their stronger ties to administrative infrastructure or grant reporting mechanisms. The lack of monitoring mechanisms for practices such as preprints and material sharing indicates a potential disconnect between funder expectations and enforcement, raising questions about the effectiveness of current policies in promoting sustained behavior change among researchers.

Despite variable monitoring, many funders provide important support mechanisms to facilitate implementation. Financial assistance to cover related costs was more frequent for open access (86%), data sharing (50%), and PPI (50%), highlighting funders' recognition of cost barriers for these practices. Guiding materials and infrastructure, including repositories and technical platforms, were also frequently made available for data sharing, code sharing, and data management plans, supporting researchers in meeting policy requirements. However, formal or informal training opportunities were less consistently reported, highlighting a notable gap. Given that prior surveys^8^ have identified a lack of training and resources as key barriers to open science uptake in cardiovascular research, increasing capacity‐building initiatives may be critical to achieving more widespread adoption.

This limited and uneven adoption of open science reflects a lack of coordination that could limit the global cardiovascular research community from fully benefiting from open science. Given that cardiovascular research is inherently international, investigators in any single region rely on access to publications, data, and protocols produced worldwide. Without harmonized policies and consistent enforcement, the potential for open science to accelerate discovery and improve patient outcomes remains unrealized.

Open science promotes equitable access to knowledge, fosters transparency, and helps prevent or detect research misconduct.^18^, ^19^, ^20^, ^21^ In a climate where public trust in science has been challenged,^22^ open science can be a tool to maintain institutional and public trust.^23^, ^24^ Funders occupy a unique position as gatekeepers of research quality, able to set clear expectations for open science practices that support scientific validation and broader scrutiny of research outputs. Strong, publicly accessible policies, combined with monitoring and support, signal a commitment to accountability and provide incentives for the research community to adopt best practices. In this way, funders not only shape the behavior of their grantees but also influence the wider cardiovascular research ecosystem, fostering a culture of openness that could meaningfully advance cardiovascular health globally.

### Limitations

This study has several strengths, including its novel focus on cardiovascular research funders, a systematic and rigorous approach to identifying and analyzing policies, and an assessment of a broad range of open science practices within a single review. However, some limitations must be acknowledged. Our search was limited to publicly available documents, excluding those accessible only behind password‐protected platforms such as grant application portals. Many funders make their requirements available only at these later stages. We did not contact funders to verify the completeness of the documents, including cases in which no documents were identified. As a result, the absence of publicly available documents does not necessarily indicate the absence of relevant policies, and our findings may underestimate the prevalence of open science expectations while overstating policy gaps. Nonetheless, we consider it essential that open science expectations be made publicly available and easily accessible. Clear communication of these requirements before application is crucial to ensure transparency and to allow researchers to prepare adequately.

Additionally, our funder‐level coding approach was based on the most stringent classification observed across all eligible documents, meaning that a practice was classified as required if at least 1 document mandated it, even if other documents from the same funder only recommended it or did not mention it. While this approach captures the presence of at least 1 formal requirement within a funder's policy landscape, it may overestimate the consistency and extent of open science expectations across a funder's funding mechanism. For example, a practice required specifically for clinical trials was classified as required at the funder level, even if no such requirement existed for other study designs. Readers should therefore interpret funder‐level classifications as reflecting the most stringent expectation documented, rather than a uniform expectation across all programs. The complete document‐level data set is publicly available, allowing readers to examine how requirements vary across individual documents and funding mechanisms. Finally, our searches were conducted using keywords in both English and French, and we included documents for which reliable translations could be obtained using automatic tools such as browser‐based translators available on funders' websites. Despite these efforts, documents published exclusively in other languages or lacking accessible translations may have been missed and underrepresented in our review.

## Conclusions

In conclusion, our findings suggest that cardiovascular funders are engaging with open science but face challenges in translating policies into practice. The observed gaps in monitoring and support highlight opportunities for funders to strengthen their policies by integrating clearer compliance mechanisms and expanding resources for researchers. Coordinated efforts among funders, aligned with evolving best practices and researcher needs, will be essential to fostering a culture of transparency and reproducibility that can accelerate discovery and ultimately improve cardiovascular research findings.

Future work should explore how funder policies interact with institutional and researcher‐level factors to influence open science behaviors in cardiovascular research. For example, if a funder mandated data sharing but the grantee's institution did not, this might be seen as a barrier to implementing the practice. Better communication between funders and research‐performing organizations will likely foster greater uptake and implementation of open science practices. Additionally, qualitative research engaging key interest holders could help identify practical barriers and facilitators to policy implementation, informing more tailored and effective interventions. As open science continues to evolve, funders' roles as leaders and enablers will remain critical in shaping a more open, collaborative, and trustworthy cardiovascular research ecosystem. If cardiovascular research wishes to reap the benefits afforded by open science, funders will want to ensure that they set policies; monitor implementation; and provide resources, training, and guidance for researchers.

## Sources of Funding

Funding for this project is provided by the Heart and Stroke Foundation of Canada.

## Disclosures

K.D.C. is the Co‐Chair of the Declaration on Research Assessment (DORA).

## Supporting information

Table S1

## References

[1] UNESCO . UNESCO recommendation on open science. 2021. doi: 10.54677/MNMH8546.

[2] White House Office of Science and Technology Policy . Memorandum for the heads of executive departments and agencies [Internet]. 2022. Report No. https://www.whitehouse.gov/wp‐content/uploads/2022/08/08‐2022‐OSTP‐Public‐Access‐Memo.pdf.

[3] Cobey KD , Liu PP . A call to embrace a culture of openness in cardiovascular research. Eur Heart J. 2022;43:2261–2263. doi: 10.1093/eurheartj/ehac189 35511501 PMC9209008

[4] Heckerman GO , Tzng E , Campos‐Melendez A , Ekwueme C , Mueller A . Transparency of research practices in cardiovascular literature. eLife. 2025;14:e81051. doi: 10.7554/eLife.81051 40135605 PMC12068865

[5] Williams JL , Chu HC , Lown MK , Daniel J , Meckl RD , Patel D , Ibrahim R . Weaknesses in experimental design and reporting decrease the likelihood of reproducibility and generalization of recent cardiovascular research. Cureus. 2022;14:e21086. doi: 10.7759/cureus.21086 35155034 PMC8825449

[6] Ioannidis JPA , Greenland S , Hlatky MA , Khoury MJ , Macleod MR , Moher D , Schulz KF , Tibshirani R . Increasing value and reducing waste in research design, conduct, and analysis. Lancet. 2014;383:166–175. doi: 10.1016/S0140-6736(13)62227-8 24411645 PMC4697939

[7] Glasziou P , Altman DG , Bossuyt P , Boutron I , Clarke M , Julious S , Michie S , Moher D , Wager E . Reducing waste from incomplete or unusable reports of biomedical research. Lancet. 2014;383:267–276. doi: 10.1016/S0140-6736(13)62228-X 24411647

[8] Cobey KD , Alayche M , Saba S , Barnes NY , Ebrahimzadeh S , Alarcón E , Hibbert B , Moher D . Cardiology researchers' practices and perceived barriers to open science: an international survey. Open Heart. 2024;11:e002433. doi: 10.1136/openhrt-2023-002433 38233041 PMC10806507

[9] Roth GA , Mensah GA , Johnson CO , Addolorato G , Ammirati E , Baddour LM , Barengo NC , Beaton AZ , Benjamin EJ , Benziger CP , et al. Global burden of cardiovascular diseases and risk factors, 1990–2019: update from the GBD 2019 study. J Am Coll Cardiol. 2020;76:2982–3021. doi: 10.1016/j.jacc.2020.11.010 33309175 PMC7755038

[10] Tan AC , Webster AC , Libesman S , Yang Z , Chand RR , Liu W , Palacios T , Hunter KE , Seidler AL . Data sharing policies across health research globally: cross‐sectional meta‐research study. Res Synth Methods. 2024;15:1060–1071. doi: 10.1002/jrsm.1757 39275943

[11] Global Cardiovascular Research Funders Forum . About us [Internet]. [cited 2026 Apr 30]. https://www.gcrff.org/about‐us

[12] Tricco AC , Lillie E , Zarin W , O'Brien KK , Colquhoun H , Levac D , Moher D , Peters MDJ , Horsley T , Weeks L , et al. PRISMA extension for scoping reviews (PRISMA‐ScR): checklist and explanation. Ann Intern Med. 2018;169:467–473. doi: 10.7326/M18-0850 30178033

[13] Braun V , Clarke V . Thematic analysis. APA handbook of research methods in psychology, Vol 2: Research designs: Quantitative, qualitative, neuropsychological, and biological. American Psychological Association; 2012:57–71. doi: 10.1037/13620-004

[14] Powell‐Wiley TM . Centering patient voices through community engagement in cardiovascular research. Circulation. 2023;147:105–107. doi: 10.1161/CIRCULATIONAHA.122.061112 36622908 PMC10493187

[15] Hendriks JM , McGreavy P , Fredericks S , Jones I , Ainslie J , Gomes A , Sanders J . Patient and public involvement in the management and prevention of cardiovascular disease: a statement of the Association of Cardiovascular Nursing and Allied Professions of the ESC and the ESC patient forum. Eur J Cardiovasc Nurs. 2026:zvaf191. doi: 10.1093/eurjcn/zvaf191 41485473

[16] Bramley P , Bird C , Badgett R , DeVito NJ . Patterns of preregistration and publication of trials in Cochrane systematic reviews of interventions. J Clin Epidemiol. 2025;187:111958. doi: 10.1016/j.jclinepi.2025.111958 40902861

[17] Hardwicke TE , Wagenmakers EJ . Reducing bias, increasing transparency and calibrating confidence with preregistration. Nat Hum Behav. 2023;7:15–26. doi: 10.1038/s41562-022-01497-2 36707644

[18] Nahm FS . Data sharing and research integrity: lessons from a recent retraction. Korean J Pain. 2025;38:1–3. doi: 10.3344/kjp.24391 39743316 PMC11695253

[19] Lumbard H , Routledge D . Open science and transparency are our strongest tools in the fight against fraudulent publishing activities. PLoS Med. 2025;22:e1004774. doi: 10.1371/journal.pmed.1004774 41004541 PMC12483235

[20] Bouter L . Research misconduct and questionable research practices form a continuum. Account Res. 2023;31:1–5. doi: 10.1080/08989621.2023.2185141 36866641

[21] Armond ACV , Cobey KD , Moher D . Research integrity definitions and challenges. J Clin Epidemiol. 2024;171:111367. doi: 10.1016/j.jclinepi.2024.111367 38642717

[22] Cologna V , Mede NG , Berger S , Besley J , Brick C , Joubert M , Maibach EW , Mihelj S , Oreskes N , Schäfer MS , et al. Trust in scientists and their role in society across 68 countries. Nat Hum Behav. 2025;9:713–730. doi: 10.1038/s41562-024-02090-5 39833424 PMC7617525

[23] Iordanou K , Antoniou A , De Vos M . Public Trust in Science: a systematic literature review. J Acad Ethics. 2026;24:56. doi: 10.1007/s10805-026-09732-5

[24] Rosman T , Bosnjak M , Silber H , Koßmann J , Heycke T . Open science and public trust in science: results from two studies. Public Underst Sci. 2022;31:1046–1062. doi: 10.1177/09636625221100686 35699352 PMC9630960

