# Supplementary material for: Funders' Expectations for Open Science in Cardiovascular Research: A Scoping Review of the Largest Cardiovascular Research Funders
Source: J Am Heart Assoc. 2026 May 6;15(10):e048584. doi: 10.1161/JAHA.125.048584 (PMC13279354; doi:10.1161/JAHA.125.048584)
Supplement: Supplementary file 1 — Table S1 [file JAH3-15-e048584-s001.pdf]

Supplemental Material

Table S1. Comparison of open science expectations between all funders and funders with retrieved documents

Open science practice	Funders where the practice is required or recommended (n)	% of all funders (n=12)	% of funders with documents retrieved (n=9)
Data sharing	8	67%	89%
Open access	7	58%	78%
Prospective registration	6	50%	67%
Public and patient involvement	6	50%	67%
Data management plans	5	42%	56%
Preprint	4	33%	44%
Code sharing	3	25%	33%
Use of ORCID	3	25%	33%
Material sharing	2	17%	22%
Reporting guidelines	2	17%	22%
Rigor and reproducibility	1	8%	11%
Open peer review	0	0%	0%

Percentages are calculated using two denominators: all funders in the Global Cardiovascular Research Funders Forum (n=12), and only funders with retrievable eligible documents (n=9).
